# Psychopathic Traits Associate With Later Schizophrenia

**DOI:** 10.1111/acps.70027

**Published:** 2025-08-25

**Authors:** Olli Vaurio, Jari Tiihonen, Markku Lähteenvuo, Johannes Lieslehto

**Affiliations:** ^1^ Department of Forensic Psychiatry, Niuvanniemi Hospital University of Eastern Finland Kuopio Finland; ^2^ Department of Clinical Neuroscience, Division of Insurance Medicine Karolinska Institutet Stockholm Sweden; ^3^ Center for Psychiatry Research Stockholm City Council Stockholm Sweden; ^4^ Institute for Molecular Medicine Finland University of Helsinki Helsinki Finland

**Keywords:** epidemiology, forensic psychiatry, psychopathy, psychotic disorders/schizophrenia, substance use disorders

## Abstract

**Introduction:**

Despite well‐known diagnostic and neurobiological overlaps between psychopathic traits and schizophrenia, it has remained unclear whether psychopathic traits increase the risk for later schizophrenia. Former studies have proven only a weak correlation between psychopathy and DSM axis I diagnoses.

**Methods:**

We combined data from individuals who underwent forensic psychiatric evaluations (FPEs) at Niuvanniemi Hospital between 1984 and 1993 with the records from the Care Register for Health Care to examine the relationship between psychopathic traits, measured by the Psychopathy Checklist‐Revised (PCL‐R), and the development of schizophrenia following the evaluation. We conducted survival analyses using Kaplan–Meier estimates and Cox proportional hazards models, with a follow‐up period of up to 40 years. Mortality data were obtained from the National Death Registry. Statistical analyses were adjusted for age, sex, criminal responsibility, and substance abuse disorder at the time of the FPE.

**Results:**

The study included 341 individuals (278 males [81.51%] and 63 females [18.49%], mean [SD] age 33.52 [11.49]) who were adults, criminally responsible, and did not have a psychotic illness, severe mental disability, or brain damage at FPE. Compared to individuals with total PCL‐R scores less than or equal to 10, those with scores of 11–24 (adjusted hazard ratio [aHR] = 5.30, 95% CI = 1.21–23.25) and 25 or higher (aHR = 9.33, 95% CI = 2.04–42.76) had a significantly higher risk of later hospitalization due to schizophrenia. Also, individuals classified as psychopathic (PCL‐*R* ≥ 25) had a significantly higher risk of developing schizophrenia compared with those classified as non‐psychopathic (PCL‐*R* < 25): aHR = 2.37, 95% CI =1.17–4.80. A total of 20% of psychopaths developed schizophrenia over the follow‐up.

**Conclusions:**

The novel results suggest that there is a link between higher PCL‐R scores and a higher risk of later‐life schizophrenia outbreak among non‐psychotic individuals undergoing FPE. Multiple factors can explain the finding, including substance use and mutual risk factors.

**Summary**

- Significant outcomes
  ○ This study combined data from individuals who underwent forensic psychiatric evaluations (FPEs) with the records from the Care Register for Health Care to examine the relationship between psychopathic traits, measured by the Psychopathy Checklist‐Revised (PCL‐R), and the development of schizophrenia following the evaluation.
  ○ The results suggest that there is a link between higher PCL‐R scores and a higher risk of later‐life schizophrenia outbreaks among non‐psychotic individuals undergoing FPE.
  ○ Multiple factors can explain the finding, including substance use and mutual risk factors.
- Limitations
  ○ The lack of access to detailed patient records concerning the clinical presentation of schizophrenia limits our ability to ascertain the full spectrum of symptomatology.
  ○ The unavailability of longitudinal data on substance use following the FPE further constrains the interpretation of its potential impact on the observed outcomes.

## 1 Introduction

Psychopathy, a severe manifestation of antisocial personality disorder typified by callous affect, egocentricity, and pronounced criminal versatility, is observed in approximately 1% of the general population [1], and disproportionately prevalent, reaching 16%–25%, within correctional settings [2]. A psychopath is a person scoring 30 (or 25 in Europe) points or more in the Psychopathy Checklist‐Revised (PCL‐R), but psychopathic traits occur in various degrees in the general population also [3]. In contrast, schizophrenia, the most common chronic psychotic disorder affecting nearly 1% of the population and over 23 million people worldwide [4], is traditionally conceptualized as an axis I condition. Although some previous investigations reported only a weak correlation between psychopathy and DSM axis I disorders (with the notable exception of substance use disorders) [5], other cohorts have hinted at a more complex interplay. There is evidence that conduct disorder, one of the most common childhood psychiatric diagnoses, can predict later psychiatric morbidity, including both psychopathy and schizophrenia [6, 7]. Also, Schizophrenia patients with a history of childhood conduct disorder are more likely to have Antisocial Personality Disorder and they obtain higher psychopathy scores than those without previous antisocial behaviour [8]. Individuals with intermediate scores on the Psychopathy Checklist‐Revised (PCL‐R) have exhibited sporadic features of paranoid schizophrenia and schizotypal personality alongside neuropsychological abnormalities [9] Subsequent studies have underscored an association between elevated psychopathic traits and paranoid personality disorder [10, 11, 12], with previous evidence suggesting that schizophrenia is markedly overrepresented among individuals with antisocial personality traits, up to 6.9 times more common in males and 11.8 times in females [13].

Neuroimaging studies have implicated structural similarities underpinning both schizophrenia and psychopathic traits. A recurrent finding in these disorders is the loss of prefrontal cortical gray matter, with deficits in frontal lobe volumes [14] documented in psychotic disorders and antisocial variants alike [15, 16]. In psychopathy, these alterations may partially account for impulsivity and impaired moral judgment, whereas in schizophrenia, they are linked to compromised working memory, abstract reasoning, and executive functions, as well as the emergence of auditory and visual hallucinations, with the magnitude of gray matter loss correlating to the severity of positive symptoms [14, 17]. Complementary to these gray matter changes, white matter integrity is also disrupted; for example, reduced integrity of the right uncinate fasciculus, a key connector between the ventromedial prefrontal cortex and anterior temporal lobe, has been associated with the interpersonal deficits characteristic of psychopathy, including pathological lying and manipulativeness [18]. Moreover, deficits in the microstructural integrity of frontotemporal white matter are more pronounced in psychopathic individuals with severe impulse control issues [19], and somewhat similar disintegration of white matter tracts is observed in schizophrenia [20]. Also, our recent machine learning investigation demonstrated that a neuroimaging‐based model trained to distinguish patients with schizophrenia from controls erroneously classified about half of non‐psychotic individuals with antisocial personality disorder, with such misclassifications occurring most frequently in those with markedly impaired functional outcomes [21]. Also, changes in several monoamine‐related genes have been implicated in both disorders: monoamine oxidase A (MAOA) [22, 23], catechol‐O‐methyltransferase (COMT) [24, 25], dopamine receptor 2 genes (DRD2) [26, 27], and serotonin‐transporter‐linked promoter region gene (5‐HTTLPR) [28, 29].

Despite the abovementioned substantial diagnostic and neurobiological overlaps, it still remains uncertain whether early psychopathic traits predispose individuals to later schizophrenia. The temporal trajectory linking psychopathic features to subsequent schizophrenia remains equivocal, owing in part to the inherent complexities of concurrently investigating these interrelated phenomena. Psychopathy, like personality disorders in general, is not diagnosed formally until the age of 18, although antecedents of psychopathic traits can be observed from early childhood onwards [6]. While the similar childhood conduct disorders and also prodromal symptoms can predict schizophrenia [7], the main symptoms generally emerge between 21–25 years in males and 25–30 years in females, with some cases manifesting over a substantially protracted period [30]. Moreover, the heterogeneous trajectories of psychotic illness, from abrupt onset to gradual, subtle progression, necessitate long‐term follow‐up spanning several decades, further complicating longitudinal research.

### 1.1 Aims of the Study

We examined a naturalistic cohort, monitored for up to 40 years and comprising individuals undergoing forensic psychiatric evaluation (FPE), to ascertain whether psychopathic traits serve as predictors of subsequent schizophrenia. Since substance‐use disorders may mediate this association, we also evaluated the relationship between psychopathic traits and later substance‐use‐related hospitalizations as a secondary outcome.

## 2 Methods

### 2.1 Study Design and Data Acquisition

We combined data from individuals who underwent forensic psychiatric evaluations (FPEs) at Niuvanniemi Hospital between 1984 and 1993 with records from the Care Register for Health Care to examine the relationship between psychopathic traits, measured by the Psychopathy Checklist‐Revised (PCL‐R) [3], and the development of schizophrenia (as measured with hospitalization) following the evaluation. As is the standard policy in Finland, the offenders were ordered to FPE by the court in the preliminary hearing. Subjects were charged with felonies, mostly homicides and other violent crimes. The procedure of forensic psychiatric evaluation in Finland has been practically unchanged since the 1980s. It is an exceedingly thorough medical investigation lasting up to 2 months and covers comprehensively both psychiatric and somatic matters. Information is gathered from numerous sources, including schools, hospitals, family members, police, military, and former employers. A psychiatrist and a psychologist interview offenders multiple times, and the vast amount of written information is then compared to the examinees' narrative to evaluate its trustfulness and realism. The Finnish criminal law states that a person is not criminally responsible if, at the time of the crime, he/she was not able to understand the factual nature or unlawfulness of his/her act, or his/her ability to control his/her behavior was decisively weakened due to mental illness, severe mental deficiency, a serious mental disturbance, or a serious disturbance of cognition. If a person's ability to understand or control his/her behavior is not crucially albeit significantly weakened, the person is regarded as having diminished responsibility, which may mitigate his/her sentence. Individuals who were legally accountable or had diminished responsibility were sentenced to prison, whereas those who were not guilty by reason of insanity or intellectual disability were committed to involuntary treatment in a mental hospital or institution for the intellectually disabled [31].

From the initial cohort, we excluded those not criminally responsible, that is, individuals with a severe mental illness (ICD‐10 diagnosis F20–F29), intellectual disability (IQ < 69)or organic psychotic disorder (F06.x). From the 15 to 30 page FPE files, we collected information on age, sex, type of index crime, diagnoses, and possible substance abuse. Each of the 20 PCL‐R items was scored 0, 1, or 2 where the higher value represents higher correlation with each psychopathic feature with a maximum score of 40 points. PCL‐R is also validated when performed retrospectively with only written documents as a source [32]. A typical European standard of 25 points was used as a cutoff point instead of 30 points used in the United States [33]. Participants were followed up through comprehensive national registries from 1984 to 2023. Mortality data were obtained from the National Death Registry, and hospitalization data were retrieved from the Care Register for Health Care: primary outcome, schizophrenia‐related hospitalizations (ICD‐8 and ICD‐9 codes 295.0–295.9, excluding 295.5; ICD‐10 codes F20 and F25); secondary outcome, substance‐use–related hospitalizations (ICD‐8 codes 291.0–292.9; ICD‐9 codes 291.0–292.9; ICD‐10 codes F10–F19). Unfortunately, the criminal records from the early 1990s were not available, and therefore reoffending could not be assessed. The linkage was done by Findata, and the research team received pseudonymized data.

### 2.2 Statistical Analyses

Our primary outcome was schizophrenia‐related hospitalization, and we investigated total PCL‐R scores [3] (primary analysis) and Factors 1 and 2 (secondary analyses). Factor 1 focuses on interpersonal and affective traits: superficial charm, grandiosity, pathological lying, manipulativeness, shallow affectivity, absence of guilt or empathy, and failure to accept responsibility. Factor 2 assesses the antisocial lifestyle and includes items: need for stimulation, parasitic lifestyle, poor behavioral control, early behavioral problems, lack of realistic, long‐term goals, impulsivity, irresponsibility, juvenile delinquency, revocation of conditional release, and criminal versatility. The secondary outcome was substance use‐related hospitalizations. The PCL scores were assessed from the FPE files by a single person (O.V.) with proven interrater reliability (interclass correlation coefficient, ICC 0.89). All statistical analyses were conducted using R version 4.4.1, with *survival* and *survminer* packages [34]. Survival analyses were conducted using Kaplan–Meier estimates and Cox proportional hazards models, with a follow‐up period of up to 40 years. The endpoint of the follow‐up was the hospitalization due to schizophrenia (primary outcome) or substance use (secondary outcome) after FPE, death, or end of available follow‐up. The Cox proportional hazard model for schizophrenia‐related hospitalization was adjusted for age, sex, criminal responsibility (full or diminished), missing PCL‐R items, and substance abuse disorder at the time of the FPE. The model for substance‐use–related hospitalization included the same covariates, excluding baseline substance‐use disorder. The proportional hazards assumption was evaluated using Schoenfeld residuals, and no violations were detected in any of the tested models. The results from Cox proportional hazard models were presented as adjusted hazard ratios (aHR) with 95% confidence intervals (95% CI).

## 3 Results

### 3.1 Demographic Results

**FIGURE 1:**
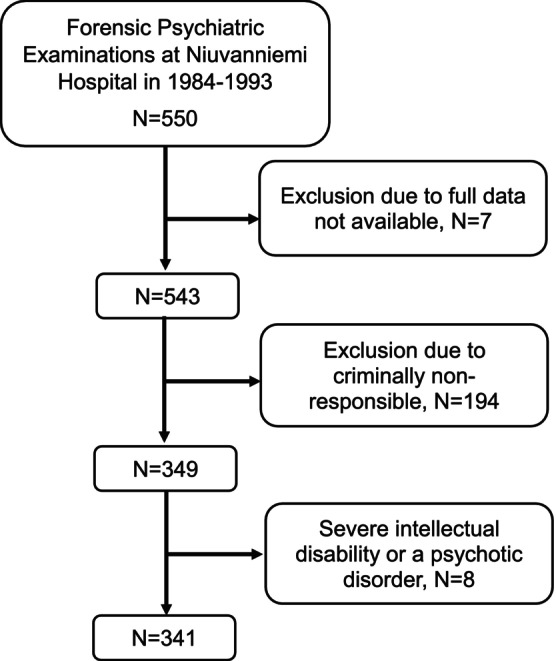
A flowchart of the study sample.

This study encompassed 341 individuals (278 males [81.51%] and 63 females [18.49%], mean [SD] age 33.52 [11.49] years, range 16–71 years, mean [SD] follow‐up 16.5 [11.49] years) who were judged criminally responsible or diminishedly responsible, did not exhibit psychosis, and were free from severe intellectual disability at FPE. The majority of participants were diagnosed with at least one personality disorder and substance use disorder, and had committed a violent index offense prompting FPE. The total PCL‐R scores ranged from 0 to 40; scores were not normally distributed: Shapiro Wilk's normality test was *W* = 0.96, *p* value < 0.0001. Of the 341 individuals, 268 (78.59%) had alcohol use disorder (AUD), and 22 of them had some additional SUD comorbidity (8.21%). Detailed demographic characteristics are presented in Table 1, and the sample formation process is illustrated in Figure 1.

**TABLE 1:** Demographic characteristics of the study sample.

Variable	Sample (*N* = 341)
Females [*N* (%)]	63 (18.49%)
Age [*M* (SD)]	33.52 (11.49)
PCL‐R total scores [*M* (SD)]	17.84 (10.42)
Any personality disorder [*N* (%)]	256 (75.07%)
Alcohol use disorder	268 (78.59%)
Perpetrator of a violent crime [*N* (%)]	256 (75.07%)
Diminished responsibility [*N* (%)]	184 (53.96%)

### 3.2 Statistical Results

**FIGURE 2:**
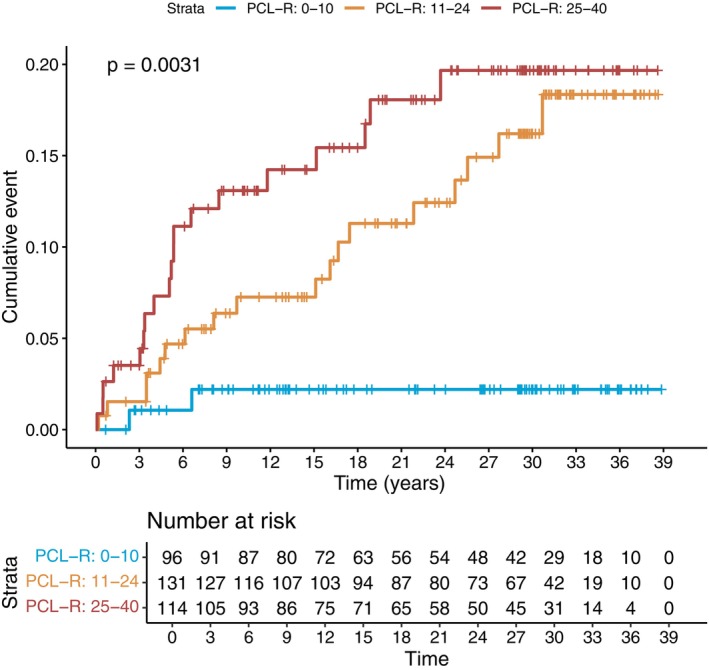
Reversed Kaplan–Meier plot for the conversion to schizophrenia post‐FPE stratified by the PCL‐R scores.

Compared to individuals with total PCL‐R scores less than or equal to 10, those with scores of 11–24 (aHR = 5.30, 95% CI: 1.21–23.25) and 25 or higher (aHR = 9.33, 95% CI: 2.04–42.76) had a significantly higher risk of later hospitalization due to schizophrenia. Also, individuals classified as psychopathic (i.e., PCL‐*R* ≥ 25) had a significantly higher risk of developing schizophrenia compared with those classified as non‐psychopathic (PCL‐*R* < 25): aHR = 2.37, 95% CI = 1.17–4.80. Figure 2 displays a reversed Kaplan–Meier plot, demonstrating a clear trend whereby higher PCL‐R scores were associated with an increased likelihood of conversion to schizophrenia following the FPE (log‐rank test *p* = 0.003). A total of 20% of psychopaths developed schizophrenia over the total follow‐up. Among those who developed schizophrenia over the follow‐up (*N* = 39), those who had PCL‐*R* ≥ 25 had a lower age of onset (mean [SD] age = 36.74 [12.67]) than those with PCL‐*R* < 25 (mean [SD] age = 42.00 [13.25]), although the small sample size rendered this non‐significant (*T* = −1.26, *p* value = 0.21).

**FIGURE 3:**
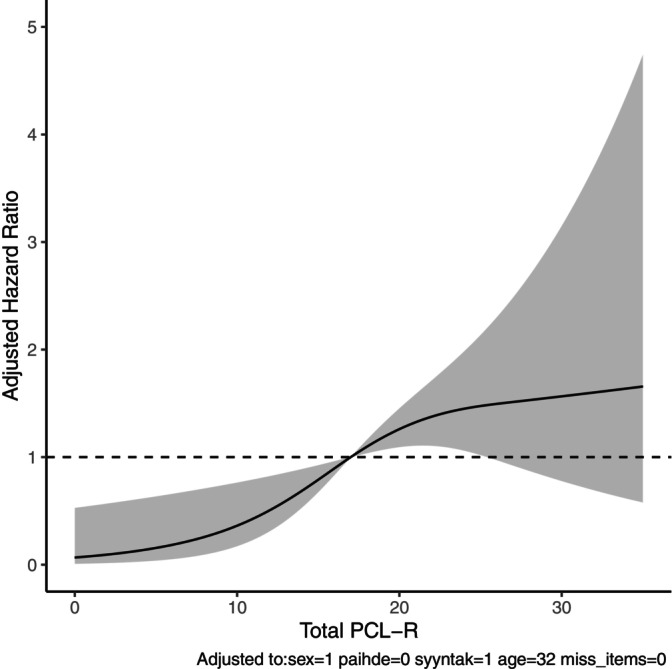
Restricted cubic spline fitting for the association between PCL‐R and schizophrenia.

We applied the restricted cubic spline method (Figure 3), using the median PCL‐R value of 17 as the reference point. A linear association was observed between PCL‐R and the risk of schizophrenia (Wald's test *p* = 0.006), with each one‐point higher PCL‐R associated with an increased risk of schizophrenia (aHR = 1.07, 95% CI = 1.03–1.11). There was no evidence of a non‐linear relationship (Wald's test *p* = 0.09). Each one‐point increment in Factor 1 was associated with an aHR of 1.13 (95% CI 1.04–1.24) for subsequent schizophrenia, with a similar association noted for Factor 2 (aHR 1.13, 95% CI 1.05–1.21).

**FIGURE 4:**
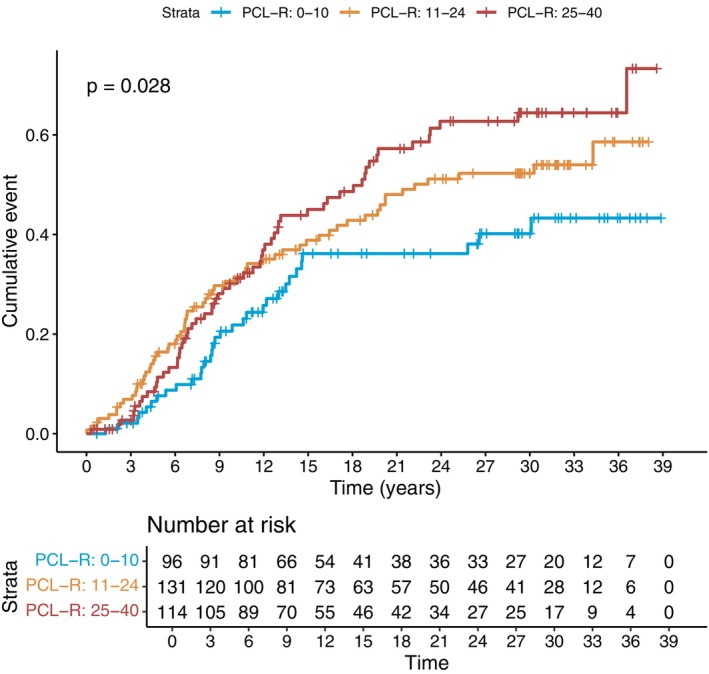
Reversed Kaplan–Meier plot for the substance abuse hospitalization post‐FPE stratified by the PCL‐R scores. As the figure illustrates, psychopathic traits are associated with higher substance abuse hospitalization after forensic psychiatric evaluation, which also partly explains the conversion from psychopathy to schizophrenia. The log‐rank test is statistically significant, *p* = 0.028.

Compared with individuals with PCL‐R scores < 10, those with scores ≥ 25 had a significantly higher risk of substance use–related hospitalization (aHR = 1.80; 95% CI = 1.11–2.90), whereas those with scores of11–24 did not (aHR = 1.45; 95% CI = 0.94–2.25). Figure 4 displays a reversed Kaplan–Meier plot, demonstrating a trend whereby higher PCL‐R scores were associated with an increased likelihood of substance abuse‐related hospitalization following the FPE (log‐rank test *p* = 0.028).

## 4 Discussion

To our knowledge, this represents the first investigation probing the relationship between psychopathic traits and subsequent schizophrenia. Employing a naturalistic cohort of offenders evaluated between 1984 and 1993 and followed for up to 40 years, our findings indicate that each incremental increase in psychopathic traits was associated with a heightened likelihood of developing schizophrenia. Remarkably, not only was the incidence of new schizophrenia cases significantly elevated, but these cases were predominantly clustered among individuals exhibiting high levels of psychopathy. A fifth of psychopaths developed schizophrenia over the total follow‐up. Although non‐significant, these individuals had a five‐year lower age of onset compared to the non‐psychopathic individuals, which is in line with the previous work [35]. This unexpected finding, which contrasts with previous research reporting only infrequent co‐occurrence of these disorders [11], invites consideration of multiple potential explanatory mechanisms.

Firstly, although psychopathy and schizophrenia are clinically distinct, one marked by enduring antisocial traits and the other by chronic psychosis, they nonetheless share several predisposing factors. Intensive research has yet to establish a definitive genetic overlap, and while each condition exhibits its own unique hereditary characteristics, both are influenced by a constellation of environmental factors. Prenatal exposures, including maternal stress [36, 37], substance use [38], smoking [39, 40], and poor nutrition [41, 42], alongside adverse postnatal experiences such as childhood maltreatment [43, 44], inconsistent upbringing, and early trauma [45, 46], contribute to an elevated risk for the development of either condition. As these disorders progress, they appear to target specific neuroanatomical regions, including the prefrontal cortex, temporal lobe, amygdala, and associated limbic circuitry. Although the resultant structural changes are generally subtle and detectable only in sizeable samples, they may partly explain the potential transition from personality disorder traits to a major psychiatric illness.

One likely pathway explaining the transition from psychopathy to schizophrenia is excessive exposure to substance use. Although the association was weaker than that observed for schizophrenia conversions, we observed a trend toward higher psychopathic traits being associated with subsequent substance use–related hospitalizations. Excessive substance use is closely associated with the antisocial lifestyle intrinsic to psychopathy [47] and is also recognized as a significant risk factor for schizophrenia [48]. In our cohort, alcohol emerged as the predominant abused intoxicant. Of the 341 individuals, 268 (78.59%) had alcohol use disorder (AUD), and 22 of them had some additional SUD comorbidity (8.21%) [49]. At study inception in the early 1990s, approximately 5% of individuals aged 15–69 had experimented with cannabis; however, this figure increased more than fourfold during the follow‐up period until the year 2022, while amphetamine use exhibited only a gradual yet steady rise [50]. Analysis of hospital registers encompassing 18,478 patients with substance‐induced psychosis in Finland revealed striking disparities: the 8‐year cumulative risk for progression to a schizophrenia spectrum diagnosis was 46% for those with cannabis‐induced psychosis and 30% for individuals with amphetamine‐induced psychosis, in contrast to a markedly lower risk of 5.0% following alcohol‐induced psychosis. Importantly, most conversions to schizophrenia spectrum diagnoses occurred within the first 3 years following the index treatment period [51].

Third, when evaluating a cohort composed mainly of antisocial individuals, the potential for symptom simulation or dissimulation must also be acknowledged. In such samples, feigned symptoms are often driven by secondary gains, such as access to social benefits, to secure accommodation, obtain psychotropic medications, or avoid criminal prosecution [52]. To safeguard diagnostic accuracy, we restricted our analyses to hospital‐confirmed schizophrenia diagnoses (ICD F20 and F25) supported by sufficient follow‐up. In the 1990s, schizophrenia was diagnosed in Finland according to ICD‐9 criteria, with a transition to ICD‐10 in 1996; per these standards, a diagnosis necessitates at least two of the following symptoms: delusions, hallucinations, disorganized speech, disorganized or catatonic behavior, and negative symptoms, persisting for a minimum of 6 months, and not attributable to acute substance use or organic causes. As forensic hospitalization in Finland can be longer than a majority of prison sentences, the concealment of psychotic symptoms (dissimulation) can be a potentially attractive strategy for offenders undergoing forensic psychiatric evaluation to avoid involuntary and possibly prolonged psychiatric treatment, and as such, it is possible that some individuals had an undetected psychotic disorder during their FPA [53]. Altogether, although some of the findings may be related to simulation or dissimulation, in general, FPAs in Finland are lengthy (2 months), and diagnoses of schizophrenia in the national registers are considered reliable. According to a large Finnish study, 80% of the core schizophrenia spectrum diagnoses in the hospital discharge registers were later approved by The Operational Criteria Checklist (OPCRIT) system [54].

These findings underscore a critical challenge in forensic psychiatric evaluations, where diagnostic precision bears significant legal consequences. Although schizophrenia and psychopathy are fundamentally distinct, certain overlapping traits can obscure their differentiation. For example, grandiosity, while typically rooted in a distorted self‐perception in psychopathy, may also be observed in schizophrenia, albeit with a divergent etiological basis [55]. Similarly, the absence of remorse, a hallmark of psychopathy, can be mimicked in schizophrenia by individuals whose delusional convictions drive aggressive behaviors without recognition of wrongdoing. Moreover, pervasive delusional thinking in schizophrenia may be misinterpreted as pathological deceit, whereas deficits in impulse control [56, 57] and blunted affect are common to both disorders [58, 59]. Yet, the absence of superficial charm, manipulativeness, and a pronounced need for external stimulation in schizophrenia, which, in contrast, characterize the psychopathic profile, further complicates the clinical picture. These nuanced symptom overlaps not only challenge diagnostic accuracy but also bear profound implications for forensic assessments, where misclassification can affect sentencing, rehabilitation, and the overall administration of justice [60].

The strengths of this study include the use of a naturalistic cohort followed for up to 40 years and the employment of rigorous diagnostic procedures, with schizophrenia diagnoses confirmed in hospital settings according to ICD criteria, thereby reinforcing the validity of our findings. Conversely, several limitations warrant consideration. Moreover, the lack of access to detailed patient records concerning the clinical presentation of schizophrenia limits our ability to ascertain the full spectrum of symptomatology. After the FPE, it is likely that several of the individuals in our sample received a schizophrenia diagnosis while being treated at mental healthcare services provided by their prisons as the registers capture these hospitalizations as well. Since we did not have access to the patient records, it is impossible to determine whether some subjects had a long duration of untreated psychosis. Finally, the unavailability of longitudinal data on substance use following the FPE further constrains the interpretation of its potential impact on the observed outcomes.

In conclusion, this observational study, with follow‐up extending up to 40 years, provides for the first time evidence that psychopathic traits are associated with the subsequent development of schizophrenia. Future investigations should ideally utilize birth cohorts that offer comprehensive data on perinatal events, childhood circumstances, and detailed health trajectories throughout follow‐up to further elucidate the complex relationship between psychopathy and schizophrenia.

## Author Contributions

**Olli Vaurio:** conceptualization, data collecting, data analysis, interpretation the results, writing – original draft, writing – review and editing. **Jari Tiihonen:** conceptualization, supervision, writing – review and editing. **Markku Lähteenvuo:** supervision, writing – review and editing. **Johannes Lieslehto:** conceptualization, data analysis, interpretation the results, supervision, writing – review and editing.

## Ethics Statement

The research project was reviewed and approved by the institutions responsible for maintaining the registers: Niuvanniemi Hospital (permission 21 Dec 2022), the Finnish National Institute for Health and Welfare (permission THL/4447/14.02.00/2022), and the University of Helsinki, Institute of Criminology and Legal Policy (permission 21 Sep 2023).

## Consent

Written informed consent from the participant's legal guardian/next of kin was not required to participate in this study in accordance with the national legislation and institutional requirements.

## Conflicts of Interest

Dr. Olli Vaurio reports no conflicts of interest. Dr. Jari Tiihonen has participated in research projects funded by grants from Janssen‐Cilag to his employing institution; he has served as a consultant to HealthCare Global Village, HLS Therapeutics, Janssen, Lundbeck, Orion Pharma, Teva, and WebMD Global; given an expert testimony to Janssen; and has received honoraria from Janssen, Lundbeck, Orion Pharma, Otsuka, and Teva; and received support for attending meetings and/or travel from Teva; all outside the submitted work. Dr. Markku Lähteenvuo is an owner and board member of Genomi Solutions Ltd. and Nursie Health Ltd., and has received honoraria from Sunovion, Orion Pharma, Janssen‐Cilag, Otsuka Pharma, Lundbeck, and Medscape, travel funds from Sunovion, and research grants from the Finnish Medical Foundation, the Emil Aaltonen Foundation, and the Finnish Cultural Foundation.

## Peer Review

The peer review history for this article is available at https://www.webofscience.com/api/gateway/wos/peer‐review/10.1111/acps.70027.

## Data Availability

The data that support the findings of this study are available on request from the corresponding author. The data are not publicly available due to privacy or ethical restrictions.
